# Sintered Indium-Tin Oxide Particles Induce Pro-Inflammatory Responses *In Vitro*, in Part through Inflammasome Activation

**DOI:** 10.1371/journal.pone.0124368

**Published:** 2015-04-13

**Authors:** Melissa A. Badding, Diane Schwegler-Berry, Ju-Hyeong Park, Natalie R. Fix, Kristin J. Cummings, Stephen S. Leonard

**Affiliations:** 1 Health Effects Laboratory Division, National Institute for Occupational Safety and Health, Morgantown, WV, 26505, United States of America; 2 Division of Respiratory Disease Studies, National Institute for Occupational Safety and Health, Morgantown, WV, 26505, United States of America; University of California Merced, UNITED STATES

## Abstract

Indium-tin oxide (ITO) is used to make transparent conductive coatings for touch-screen and liquid crystal display electronics. As the demand for consumer electronics continues to increase, so does the concern for occupational exposures to particles containing these potentially toxic metal oxides. Indium-containing particles have been shown to be cytotoxic in cultured cells and pro-inflammatory in pulmonary animal models. In humans, pulmonary alveolar proteinosis and fibrotic interstitial lung disease have been observed in ITO facility workers. However, which ITO production materials may be the most toxic to workers and how they initiate pulmonary inflammation remain poorly understood. Here we examined four different particle samples collected from an ITO production facility for their ability to induce pro-inflammatory responses *in vitro*. Tin oxide, sintered ITO (SITO), and ventilation dust particles activated nuclear factor kappa B (NFκB) within 3 h of treatment. However, only SITO induced robust cytokine production (IL-1β, IL-6, TNFα, and IL-8) within 24 h in both RAW 264.7 mouse macrophages and BEAS-2B human bronchial epithelial cells. Our lab and others have previously demonstrated SITO-induced cytotoxicity as well. These findings suggest that SITO particles activate the NLRP3 inflammasome, which has been implicated in several immune-mediated diseases via its ability to induce IL-1β release and cause subsequent cell death. Inflammasome activation by SITO was confirmed, but it required the presence of endotoxin. Further, a phagocytosis assay revealed that pre-uptake of SITO or ventilation dust impaired proper macrophage phagocytosis of *E*. *coli*. Our results suggest that adverse inflammatory responses to SITO particles by both macrophage and epithelial cells may initiate and propagate indium lung disease. These findings will provide a better understanding of the molecular mechanisms behind an emerging occupational health issue.

## INTRODUCTION

Production of indium-tin oxide (ITO) involves mixing powdered indium oxide and tin oxide in a 90:10 ratio (wt:wt), and then melding the materials into an ITO tile through a process called sintering. This method uses high temperatures to create the final product; an ITO tile that can be used to make “thin film” coatings [1, 2]. Recently, the demand for ITO has increased as its incorporation into transparent conductive coatings for touch-screen phones, liquid crystal displays (LCD), and solar panels makes it crucial in industries that manufacture electronics.

The increased production of ITO has resulted in more workers being exposed to indium-containing dusts, which has led to the recognition of a novel occupational condition, indium lung disease. The first case of indium compound-related fatal lung disease was reported in a worker that performed wet surface grinding of ITO, who developed interstitial pneumonia [3]. There were multiple indium- and tin-containing particles found in his lungs. Since then, other studies have examined the relationship between serum indium levels and resulting lung disease [4–7]. In terms of pulmonary symptoms and signs, workers exposed to ITO by inhalation develop cough, dyspnea, and abnormalities on pulmonary function tests and chest computed tomography (CT) scans [3, 6, 7].

A recent comprehensive clinical and epidemiologic analysis of 10 reported cases, including two from an ITO production facility in the United States, demonstrated that workers presented with pulmonary alveolar proteinosis (within 6–14 months of hire) or fibrotic interstitial lung disease with or without emphysema (within 2–14 years of hire) [8]. The current study and our previous study [9] have utilized particles collected from this same US facility at various stages in the ITO production process. These samples are in the micron size range and include various starting materials, particles containing ITO, and dusts generated during the reclamation of indium from leftover materials. Thus, we were able to expose cultured cells to the same particles that workers may encounter throughout the ITO production process.

Previous laboratory studies of indium-containing particles have demonstrated toxicity *in vitro* [9–11] and pulmonary inflammation in rodent models [10, 12–14]. However, how these particles initiate inflammatory responses and induce cellular damage leading to pulmonary disease remains unknown. Recent studies have implicated activation of the nod-like receptor protein 3 (NLRP3) inflammasome in the development of pulmonary fibrosis [15–17]. Inflammasome activation leads to the release of interleukin-1β (IL-1β), which unlike other cytokines requires not only transcriptional activation, but also activation of the cysteine protease caspase-1 to induce the processing and release of biologically active IL-1β. This cytoplasmic, multi-protein inflammasome complex is thought to play a role in the pathogenesis of several inflammatory diseases, some of which are particle-induced. This is due to the fact that NLRP3 acts as a sensor for a variety of different “danger” signals. For example, it can be triggered by endogenous particles, such as cholesterol crystals [18], monosodium urate [19] and fibrillar amyloid β [20], along with exogenous particles, such as crystalline silica [21, 22] and asbestos [23].

A main goal of this study was to determine whether a particular stage or process of ITO production puts workers at higher risk for developing indium lung disease, based on adverse cellular reactions to collected ITO production particles. Our group previously revealed sintered ITO (SITO) particles to be cytotoxic in cultured cells, but the mechanism behind this toxicity remains to be elucidated. Here we sought to determine whether cultured cells react to the particles in a pro-inflammatory manner and whether particle uptake affects cellular functions such as phagocytosis. Our findings suggest that particle uptake and subsequent pro-inflammatory responses may be responsible for the initiation and/or pathogenesis of pulmonary diseases that occur in indium-exposed workers.

## MATERIALS AND METHODS

### ITO production particles and reagents

The particles used in this study were collected at a United States ITO production facility from containers of feedstock materials or production processes [24]. Tin oxide (SnO_2_), unsintered ITO (UITO), and sintered ITO (SITO) are from the ITO department; and ventilation dust (VD) is from the reclamation department. The particles were extensively characterized in a previous study by our group [9]. The SnO_2_ is crystallographically pure and the other 3 samples are compound mixtures containing oxygen, carbon, indium, and tin. UITO is 46% O, 29% C, 22% In, and 2.8% Sn (atom % at particle surface by X-ray photoelectron spectroscopy). SITO is 44% O, 19% C, 24% In, and 2.7% Sn. VD is 21% O, 49% C, 12% In, and 0% Sn. The SITO particles were collected in the grinding area and had been in contact with metalworking fluid. The particles were suspended in sterile filtered 1X phosphate-buffered saline (PBS), vortexed, and diluted into cell culture media at a final concentration of 50 μg/ml by total weight. The dose was based on a previous cytotoxicity study of these particles by our group [9]. Stock suspensions of particles were prepared fresh for experiments and equal volumes of 1X PBS were used as control conditions. Crystalline silica (Min-U-sil, 5 μm) was used as a control particle in some experiments, due to its ability to activate the NLRP3 inflammasome [21–23, 25] and due its micron-range size, which is similar to the size of the ITO particles. Lipopolysaccharide from *E*. *coli* serotype 055:B5 (LPS) and Cytochalasin D were purchased from Sigma-Aldrich (St. Louis, MO). Glybenclamide and Z-YVAD-FMK were purchased from InvivoGen (San Diego, CA).

### Endotoxin analysis of particle samples

Because metalworking fluid is commonly contaminated with gram negative bacteria [26], and endotoxin is a potent immune stimulant, we examined the endotoxin content of SITO particles. Endotoxin potency of particle samples was determined using kinetic chromogenic *Limulus* amoebocyte lysate (LAL: Associates of Cape Cod, Inc., Falmouth, MA) assay. All glassware used was baked at 260° C for 2–2.5 h to render them endotoxin-free. Prior to the assay, 20 mg of particle sample or 1 ml of metal working fluid sample were extracted in TAP (triethylamine phosphate) buffer or LRW (LAL reagent water) with 0.005% Tween 20, respectively. The sample in extraction solution was sonicated for 1 h using an ultrasonic water bath sonicator (Bransonic Model 5510, Branson Ultrasonics Corp., Danbury CT) with vortexing every 15 min. All the sample extracts and control standard endotoxin (lot #134, Associates of Cape Cod, Inc., Falmouth, MA) were serially diluted in LAL reagent water, and 50 μl aliquot of each of the serial dilutions were added to a 96 well plate in duplicates. Then 50 μl of lysate reconstituted with Glucashield buffer (Associates of Cape Cod, Inc.) to block interference of (1→3)-ƅ-D-glucan was added to the standard, sample, and negative control (LRW and Glucashield buffer) wells. Immediately after the lysate was added, the plate was placed in a microplate reader (ELx808IU, BioTek Instruments, Inc., Winooski, VT) and optical density in each well at 405 nm was recorded every 30 s for 120 min. SITO particles were baked at 270° C and then retested to confirm a lack of endotoxin activity. With the kinetic data, endotoxin potency was estimated using onset time and a parallel-line estimation method [27]. The potency was reported in endotoxin unit (EU) relative to reference standard endotoxin (lot # G3E069; *E*. *coli* O113:H10 strain).

### Cell culture

The adherent mouse monocyte-derived macrophage cell line, RAW 264.7 (RAW), and the human bronchial epithelial cell line, BEAS-2B, were obtained from ATCC (Manassas, VA). RAW cells were selected for these studies because of their potential to react with and engulf particles [11, 28]. The inclusion of BEAS-2B bronchial epithelial cells in these analyses is due to the potential role of the lung epithelium in indium lung disease. Both cell lines were grown and passaged as previously described [9].

### Transmission electron microscopy

RAW and BEAS-2B cells were grown to about 70% confluency in 6 well dishes and treated with particle suspensions for 3 h. Following treatments, cells were washed twice with 1X PBS, scraped (RAW) or trypsinized (BEAS-2B), and centrifuged to pellet the cells. The samples were fixed in Karnovsky’s (2.5% gluteraldehyde, 2.5% paraformaldehyde in 0.1M Sodium Cacodylic buffer) fixative, post-fixed in osmium tetroxide, mordanted in 1% tannic acid and stained *en bloc* in 0.5% uranyl acetate. The pellets were embedded in epon, sectioned and stained with Reynold’s lead citrate and uranyl acetate. The sections were imaged on a JEOL 1220 transmission electron microscope (JEOL USA, Inc., Peabody, MA) at 10,000 times magnification using a 5 kV accelerating voltage.

### Phagocytosis assay

RAW cells were plated at 1 x 10^5^ cells/well in 96 well dishes and treated with particle suspensions for 3, 6, or 12 h. The pHrodo Red *E*. *coli* BioParticles were prepared according to manufacturer’s instructions (Molecular Probes, Carlsbad, CA), and the assay was carried out as previously described [29]. Briefly, at each time point, wells were washed and BioParticle suspensions were added to all wells, including blanks (no cells) to be subtracted from all experimental wells. Following incubation for 2 h at 37° C, plates were read at 560 nm excitation/600 nm emission to measure changes in fluorescence. Cytochalasin D at a final concentration of 10 μM was used as a positive control in certain wells to inhibit phagocytosis. All conditions were run in triplicate wells and at least three independent experiments were performed. The percentage of maximal phagocytosis was calculated as a ratio of the net experimental phagocytosis to the PBS control phagocytosis.

The assay above was also carried out with RAW cells on coverslips in 12 well dishes. However, rather than measuring fluorescence as a final step, the coverslips were washed twice, fixed for 10 min in formaldehyde, and mounted onto ultra clean glass slides (Schott Nexterion, Arlington, VA) with Fluoromount G. Conditions were performed in duplicate, and dual-mode images of cells with particles and *E*. *coli* were acquired using a CytoViva enhanced darkfield microscopy system (Aetos Technologies, Inc., Auburn, AL) integrated into an Olympus BX41 upright microscope equipped with an Olympus DP73 digital camera (Olympus, Center Valley, PA) to attain images of high-contrast particles (bright spots) against a dark background.

### Western blotting

RAW and BEAS-2B cells were grown to about 70% confluency in 6 well dishes and treated with particle suspension for 1 or 3 h. Cells were washed twice with 1X PBS, lysed with RIPA buffer (Thermo Scientific, Rockford, IL), and incubated on ice for 10 min prior to spinning for 15 min at 3000 x g. A BCA protein assay (Thermo Scientific) was used to determine protein concentration in each lysate sample, and lysates were boiled for 5 min with 5X laemmli sample buffer. SDS-PAGE was performed on prepared lysate samples, and proteins were transferred to PVDF membranes, and probed with antibodies against IκBα (1:1000; cat. no. sc-371, Santa Cruz Biotechnology, Santa Cruz, CA), stripped and re-probed for β-actin (1:8,000; cat. no. MA191399, Fisher Scientific, Pittsburgh, PA). Lysates from four independent experiments were run for each condition. Chemiluminescence detection and densitometry analysis were used to determine relative IκBα levels, normalized to actin loading controls, and then compared to PBS controls.

### Immunocytochemistry

When cells were plated for the western blot experiments, coverslips were first added to the culture dishes, so that a portion of the treated cells could also be fixed and examined for p65 nuclear translocation. Prior to the addition of RIPA buffer, coverslips were removed from wells and fixed for 10 min with methanol/acetone (1:1 vol:vol) at -20° C. Cells were washed twice with 1X PBS, and immunofluorescence was carried out using primary antibodies against NFκB subunit p65 (1:200; cat. no. PA1-14298, Thermo Scientific) and secondary Alexa 488 antibody conjugates (1:200; Invitrogen, Grand Island, NY). Coverslips were mounted onto slides with Fluoromount G, and cells were imaged using an Olympus AX70 microscope equipped with an Olympus DP73 digital camera (Olympus, Center Valley, PA).

### Enzyme-linked immunosorbent assay (ELISA)

3 x 10^5^ RAW cells/well and 1 x 10^5^ BEAS-2B cells/well were plated in 12 well dishes and were treated with particle suspensions for 24 h. LPS at 1 μg/ml was used as a positive control for cytokine production or was used to prime RAW cells for 3 h prior to particle suspension treatments (for IL-1β repeat measurements only). Media were collected from wells and frozen at -80° C. Cytokine levels were measured via BD OptEIA ELISA kits (mouse IL-6; cat. no. 555240, mouse IL-1β; cat. no. 559603, mouse TNFα; cat. no. 555268, human IL-6; cat. no. 555220, human IL-8; cat. no. 555244, BD Biosciences, San Diego, CA) according to manufacturer’s directions, except for a few changes following kit optimizations. For IL-1β, the protocol was modified such that secondary antibodies were diluted at 1:250. For the human IL-6 and IL-8 kits, primary and secondary antibodies were diluted at 1:500 and 1:1000, respectively. All conditions were run in duplicate wells and at least three to four independent experiments were performed.

### Inflammasome inhibition assays

RAW cells were plated, treated, and assayed as in the ELISA assay above, but cells were first pre-treated with either 200 μM of the ATP-sensitive K^+^ pump inhibitor Glybenclamide or 40 μM of the caspase-1 inhibitor Z-YVAD-FMK for 1 h prior to particle suspension treatments. Wells receiving Min-U-sil were used as a positive control for *in vitro* inflammasome activation. All conditions were run in duplicate wells and four independent experiments were performed.

### Caspase-1 activation

RAW cells were plated at 5 x 10^5^ cells/well in 6 well dishes, treated with SITO, LPS alone, or Min-U-sil (cells were first primed with LPS). Cells were washed, lysed, and 100 μg of lysates were assayed for their ability to cleave a fluorescent caspase-1 substrate, YVAD-AFC according to the manufacturer’s protocol (cat. no. ab39412, Abcam, Cambridge, MA). Values were normalized to PBS controls. All conditions were run in duplicate wells and three independent experiments were performed for each time point.

### Cytotoxicity Assay

RAW cells were plated at 2.5 x 10^4^ cells/well and BEAS-2B cells were plated at 5 x 10^3^ cells/well in 96 well dishes, followed by treatments with SITO, baked SITO, LPS, or Min-U-sil (after 3 h priming with LPS). Changes in cell viability at 24 and 48 h were assessed using the MultiTox-Fluor Cytotoxicity Assay according to the manufacturer’s directions (Promega, Madison, WI). A cell-permeable protease substrate, GF-AFC (glycyl-phenylalanyl-amino-fluorocoumerin), is cleaved by live cells to produce fluorescent AFC. Thus, the fluorescent signal is proportional to the number of viable cells. To ensure the fluorescent signal was due to substrate products and not autofluorescence or interference by the particles, separate wells of medium and particle suspensions were included in the plates, and these readings were subtracted from their respective wells that contained treated cells. Values were normalized to PBS controls. All conditions were run in triplicate wells and four independent experiments were performed.

### Statistical analysis

All data are represented as the mean ± standard deviation (SD) for each condition. One-way and two-way analysis of variance tests with Tukey or Dunnett’s post-tests were performed using GraphPad Prism 6 software (GraphPad Software, Inc., La Jolla, CA) for each experiment to compare the responses between groups, and statistical significance is shown when p < 0.05.

## RESULTS

### Process description and particle characteristics

The particles used in this study were collected from different areas of an ITO facility and represent various stages of the production process. Tin oxide (SnO_2_) is one of the starting materials used to manufacture ITO and contains no indium. Unsintered ITO (UITO) is a mixture of SnO_2_ and indium oxide (In_2_O_3_), which, after firing, becomes sintered ITO (SITO). The SITO particles were generated during wet grinding of the final product to customer specifications. The ventilation dust (VD) was collected from the ventilation system within the reclaim department of the facility, where indium is recycled from leftover materials. These particles were fully characterized in a previous study by our group, and were found to have mean aerodynamic diameters in the range of 0.5 to 1.2 μm [9]. Thus, these represent respirable particles in the micron size range. Varying amounts of indium were found on the particle surfaces via XPS elemental analysis with a sampling depth of about 10 nm (SnO_2_, 0%; UITO, 22%; SITO, 24%; VD, 12% indium). This study also revealed SITO to be the only particle type that was cytotoxic at the low dose of 50 μg/ml in both RAW and BEAS-2B cells within 48 h [9]. Thus, particle suspensions at this concentration were used for all experiments.

Post-sintered ITO tiles are sprayed with metalworking fluid that is used during cutting and grinding as a coolant, and in industrial settings metalworking fluids commonly become colonized by gram negative bacteria [26]. Results from a *Limulus* amoebocyte lysate (LAL) assay revealed that the SITO particles indeed contained endotoxin at 51.66 EU/mg, while the other samples contained negligible activity (**Table 1**). Given that 1 EU (endotoxin unit) per ml is estimated to represent 0.1 ng LPS per EU, and the SITO particle suspensions were diluted to a final concentration of 50 μg/ml for cell treatments, this equates to approximately 0.26 ng/ml LPS or 2.58 EU/ml. The source of the endotoxin was confirmed to be the metalworking fluids used on the ITO tiles post-sintering (via LAL assay on fluid samples from all 11 grinder machines). Thus, workers operating grinders could be exposed to aerosolized, endotoxin-containing SITO particles as opposed to contaminant-free SITO. We therefore explored the possible role of endotoxin in the toxicity of these particles, as they represent a real-life worker exposure.

**Table 1:** Endotoxin activity in particle samples.

Sample	EU/mg
SnO_2_	0.0060
UITO	0.0175
SITO	51.6631
VD	0.0034
Baked SITO*	0.0017

*baked for 1 h at 270° C.

### Particle uptake by cells and phagocytosis impairment

**Fig 1:**
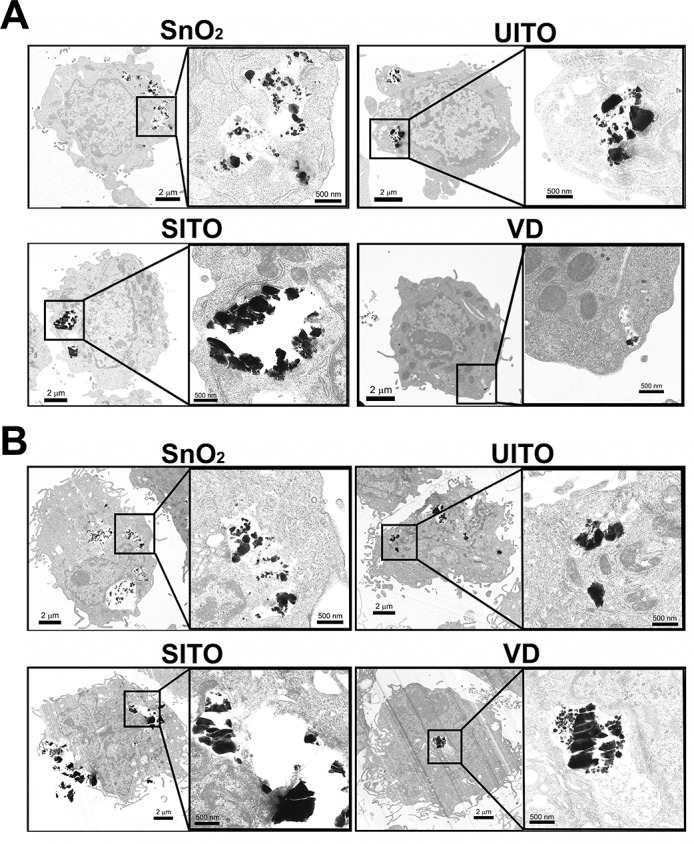
Transmission electron micrographs of cells treated with ITO production particles. Representative images of **A.** RAW and **B.** BEAS-2B cells after 3 h exposures to particle suspensions at 50 μg/ml. Images were acquired at 10,000 times magnification using a 5 kV accelerating voltage. Insets show magnified engulfed particles. Scale bars, 2 μm or 500 nm.

**Fig 2:**
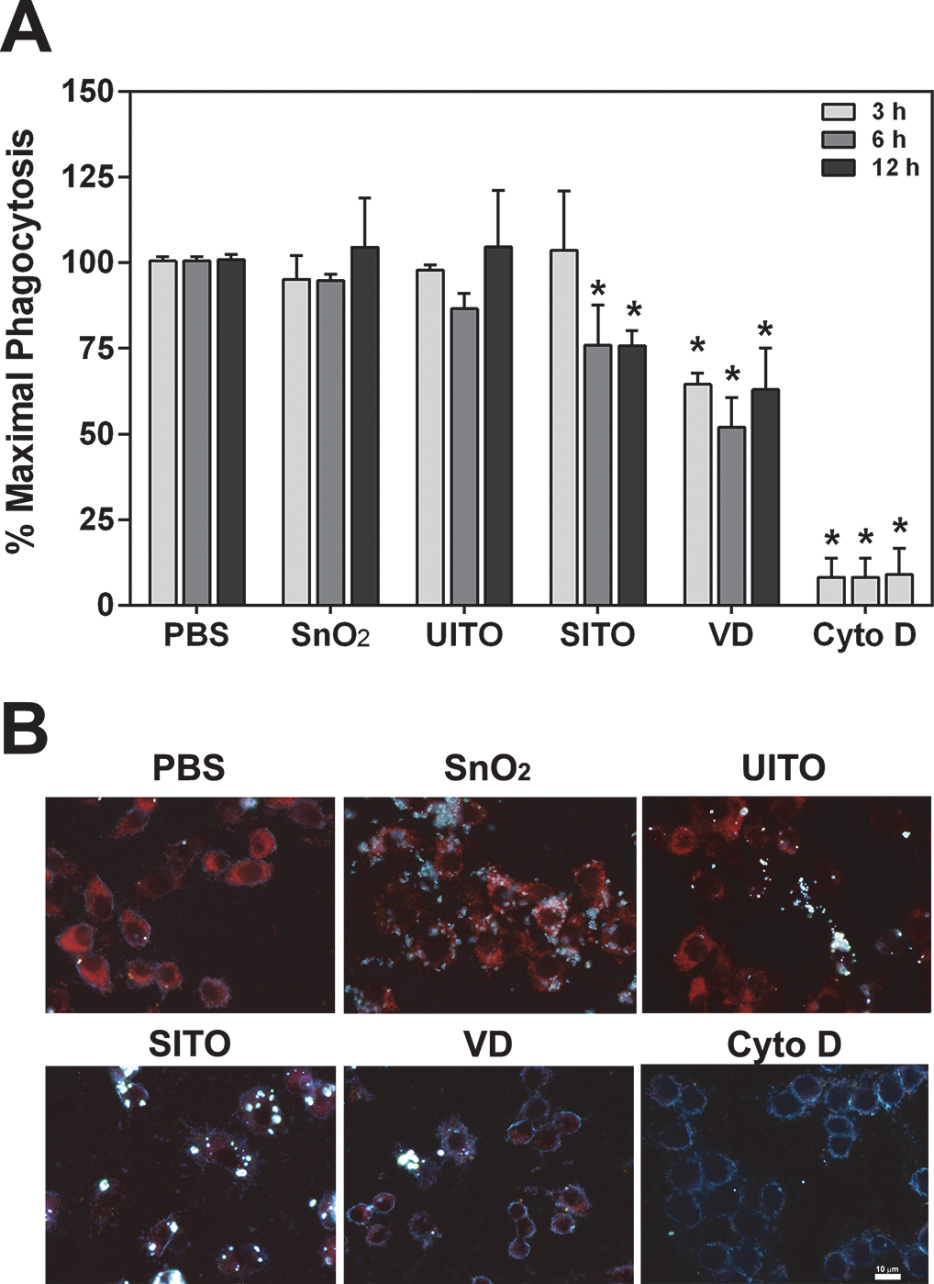
Pre-uptake of SITO or VD impairs phagocytosis. **A.** RAW cells were treated with particle suspensions for 3, 6, or 12 h or Cytochalasin D (Cyto D) 3 h. Cells were washed, pHrodo Red *E*. *coli* BioParticles were added for 2 h, then plates were read to measure changes in fluorescence. Error bars represent the mean ± SD (n = 4 for 3 h and 6 h, n = 3 for 12 h). *, p < 0.05 compared to PBS. **B.** The same as in **A**, except cells were exposed to particle suspensions for 6 h on coverslips, fixed with formaldehyde following *E*. *coli* particle incubations, and mounted onto slides. Images were acquired at 60x magnification using dual-mode fluorescence and enhanced darkfield microscopy to show phagocytosed *E*. *coli* (red) and cell-associated particles (bright spots). Scale bar, 10 μm.

Transmission electron microscopy (TEM) revealed that both RAW and BEAS-2B cells engulfed the particles within 3 h of exposure (**Fig 1**). Although not quantitative, the images of VD-treated cells appeared to have less particle uptake compared to the other three samples. We hypothesized that pre-uptake of the particles would impair the ability of macrophages to properly phagocytose pathogens. Therefore, an assay was employed using pH-sensitive *E*. *coli* particles that fluoresce upon proper phagocytosis. **Fig 2** demonstrates that when RAW cells were exposed to SITO or VD, maximal phagocytosis of subsequently added *E*. *coli* particles was significantly impaired. The effect with VD occurred more rapidly, with impairment beginning at 3 h pre-treatment, while SITO-induced reductions were first observed with a 6 h pre-treatment. Images of treated cells agree with the assay findings, in which the red fluorescence signal was drastically weaker with SITO and VD treatments (**Fig 2B**), indicating fewer *E*. *coli* particles were properly phagocytosed by cells that had taken up those particles.

### NFκB activation following ITO production particle exposures

**Fig 3:**
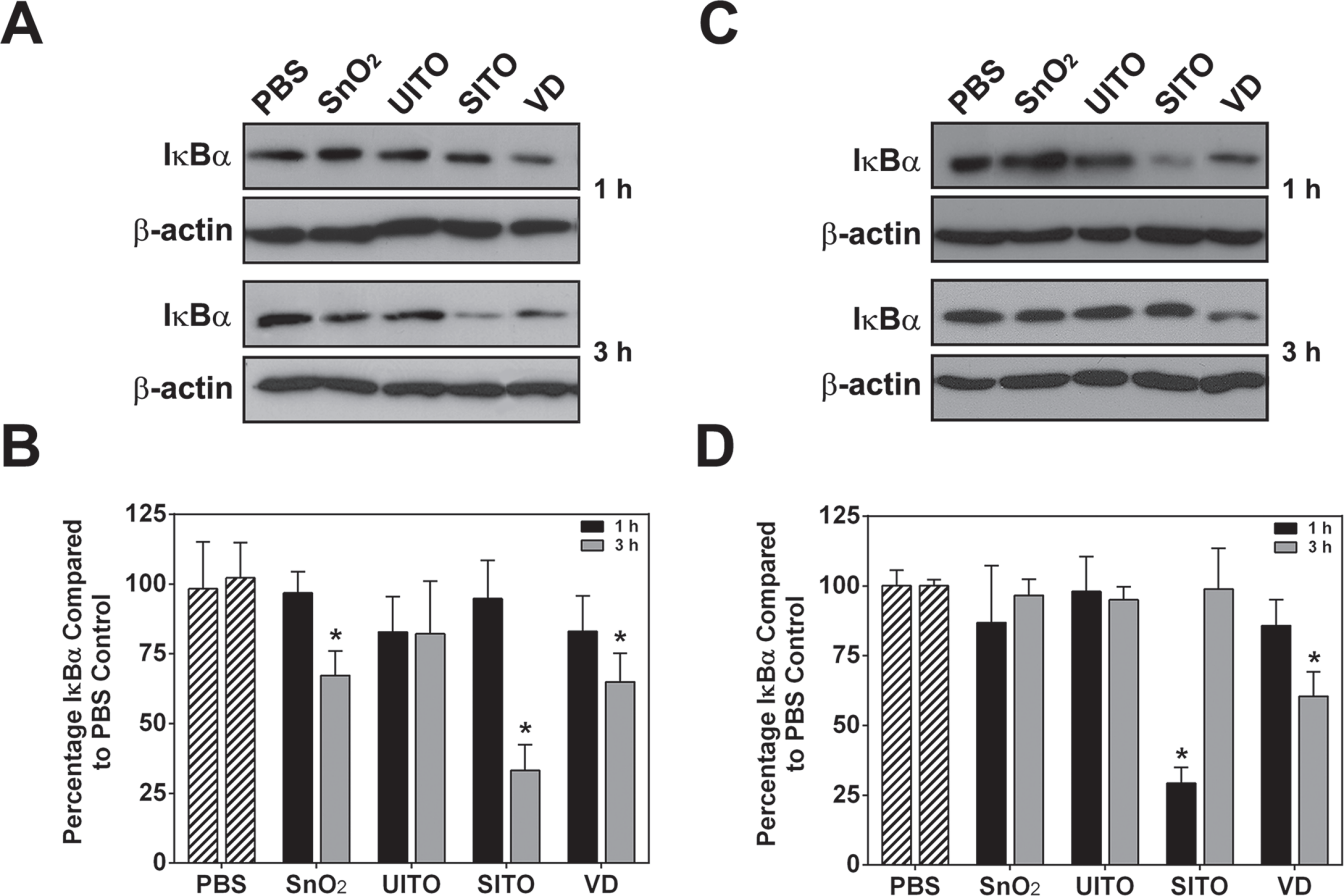
ITO production particles cause IκBα degradation. Representative western blot images of IκBα and actin from treated RAW (**A**) and BEAS-2B (**C**) cell lysates. Cells were treated with particle suspensions for 1 or 3 h, washed twice with PBS, and lysed. **B, D**. Densitometry analysis was performed and is represented as the percentage of IκBα compared to levels present in PBS control-treated lysates. Error bars represent the mean ± SD (n = 4). *, p < 0.05 compared to PBS.

**Fig 4:**
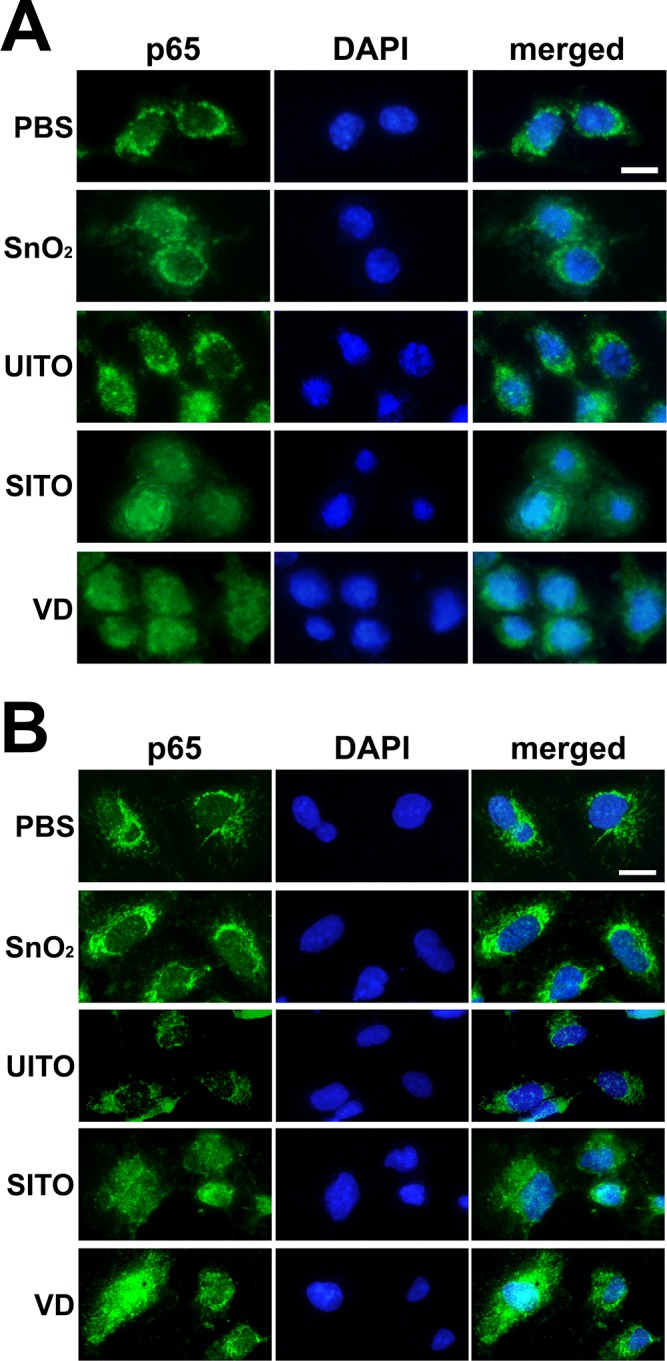
Nuclear accumulation of p65 following particle exposures. RAW (**A**) and BEAS-2B (**B**) cells were grown on coverslips and treated with particle suspensions for 3 h as in **Fig 3**. Prior to adding lysis buffer to wells, coverslips were removed, fixed, and stained with antibodies against NFκB subunit p65. Coverslips were mounted onto slides with Fluoromount G (DAPI) and sealed with nail polish. Experiments were performed in duplicate, and cells were imaged at 40x. Scale bar, 20 μm.

Given the pulmonary inflammatory response seen in animals treated with various indium-containing particles [10, 12–14], we suspected pro-inflammatory signaling pathways may be activated in cultured cells treated with ITO production particles. Thus, activation of nuclear factor kappa B (NFκB) was examined via degradation of the inhibitory protein IκBα in RAW and BEAS-2B cells. Western blotting densitometry revealed that SnO_2_, SITO, and VD caused significant IκBα protein level reductions at 3 h post-treatment in RAW cells (**Fig 3A**). Both SITO and VD also induced IκBα degradation in BEAS-2B cells (**Fig 3D**), suggesting that the particle-induced NFκB activation may not be cell type-specific. Immunocytochemistry, using antibodies against the NFκB transcription factor p65, was used to confirm nuclear accumulation of this protein following IκBα degradation. The same particles that induced IκBα degradation also had pronounced overlay of p65 with the DAPI nuclear stain in cells (**Fig 4**).

### Cytokine production in response to ITO production particles

**Fig 5:**
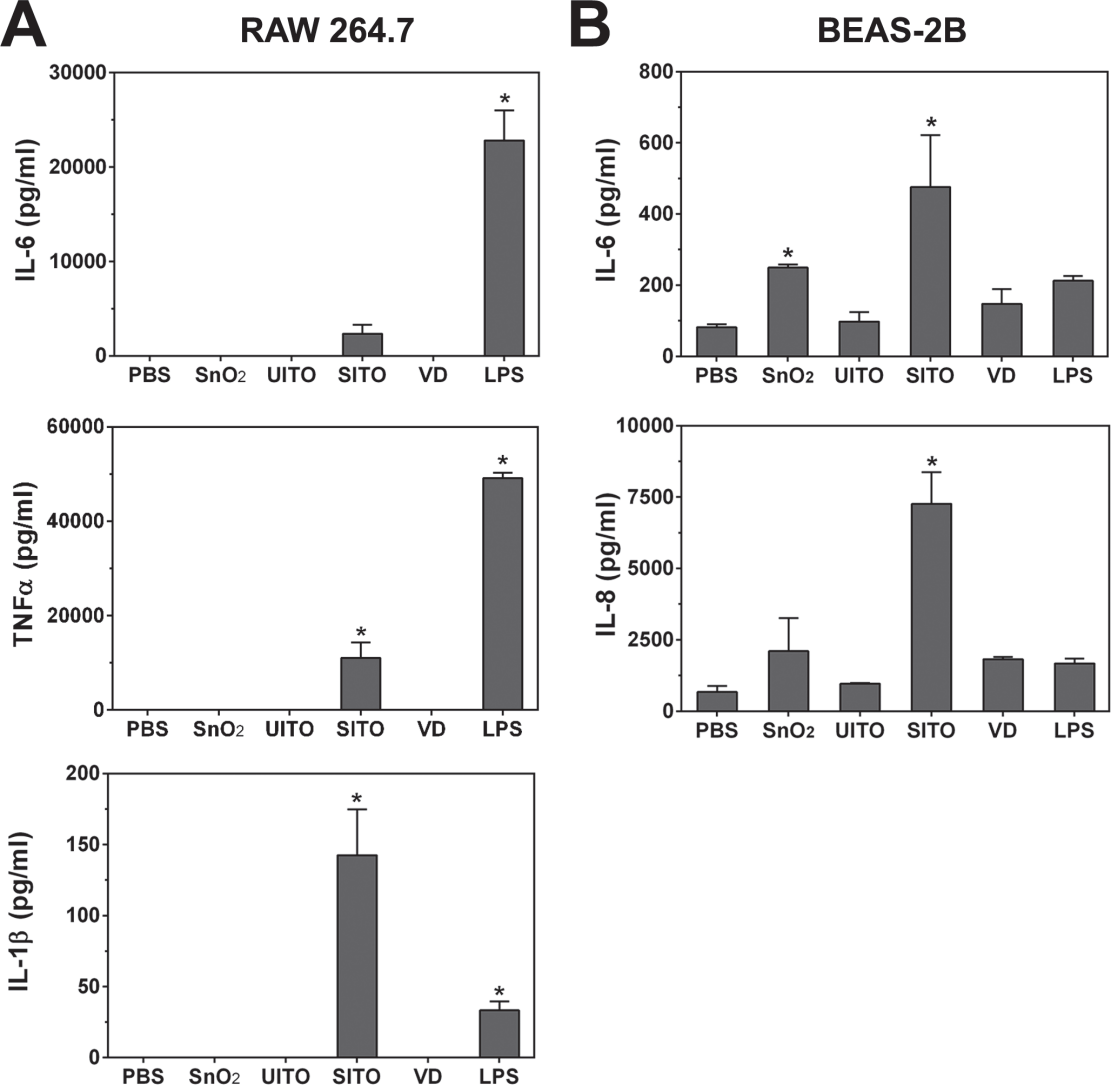
Pro-inflammatory cytokine production with particle exposures. Cells were treated with particle suspensions or 1 μg/ml of LPS as a positive control for 24 h. Media were collected, and ELISAs were run to measure levels of IL-6, TNFα, and IL-1β from RAW cells (**A**) or IL-6 and IL-8 from BEAS-2B cells (**B**). Error bars represent the mean ± SD (n = 4). *, p < 0.05 compared to PBS.

To further explore the influence of the particles on pro-inflammatory activation, various cytokines (IL-6, TNFα, and IL-1β from RAW cells and IL-6 and IL-8 from BEAS-2B cells) were measured from the culture media of cells treated for 24 h with the particle suspensions. Although SnO_2_, SITO, and VD induced NFκB activation, only SITO induced significant cytokine production in both cell lines (**Fig 5**). In BEAS-2B cells, SnO_2_ caused significant IL-6 production as well. It was not surprising that SITO was pro-inflammatory in both cell lines, given the presence of endotoxin (LPS) in this sample (**Table 1**).

### Inflammasome activation by SITO

**Fig 6:**
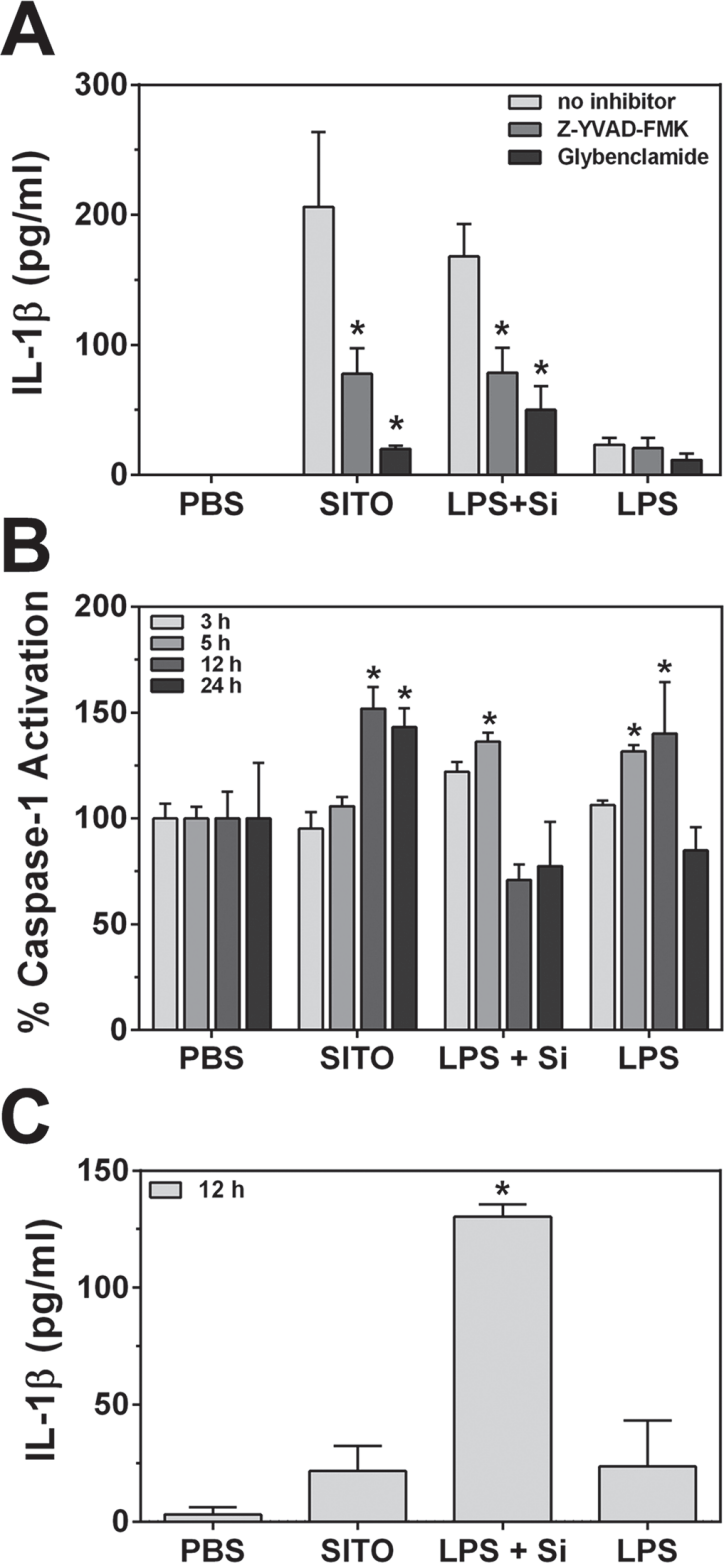
Inflammasome activation in RAW cells by SITO particles. **A.** RAW cells were pre-treated with either 40 μM Z-YVAD-FMK (caspase-1 inhibitor) or 200 μM Glybenclamide for 1 h. Cells were then treated for 24 h with SITO (50 μg/ml), LPS alone (1 μg/ml), or Min-U-sil following LPS priming (LPS + Si; 150 μg/ml). Media were collected and ELISAs were run to measure levels of IL-1β (n = 4). *, p < 0.05 compared to no inhibitor conditions. **B.** Cells were treated as in **A** (without inhibitors) for the time points listed, lysed, and 100 μg lysates were assayed for caspase-1 activity. Values were normalized to PBS controls. *, p < 0.05 compared to PBS at that time point. **C.** Media collected from (**B**) were measured for IL-1β at the 12 h time point (n = 3). Error bars represent the mean ± SD (n = 3). *, p < 0.05 compared to PBS.

The release of biologically active IL-1β from cells *in vitro* requires activation of the NLRP3 inflammasome [30]. Studies of other extracellular particles, such as cholesterol crystals [18], asbestos [23], monosodium urate [19], and crystalline silica [21, 22] report the need to first prime macrophages with LPS in order to induce IL-1β production and release. Since SITO contains low levels of LPS and induced significant IL-1β release from unprimed RAW cells, inflammasome activation was investigated. To confirm that components of the NLRP3 inflammasome were involved in this response, inhibitors were added to RAW cells prior to treatment with SITO. Glybenclamide blocks ATP-sensitive K^+^ pumps [31], while the inhibitory substrate Z-YVAD-FMK blocks caspase-1 activity. LPS priming followed by Min-U-sil treatment was used as a positive control for inflammasome activation and at a higher concentration than SITO (150 μg/ml vs. 50 μg/ml), based on concentrations used in previous studies to induce IL-1β release [21, 23, 25]. Pre-treatment of cells with either inhibitor led to significantly reduced levels of IL-1β in the culture media following a 24 h exposure of both SITO and Min-U-sil (**Fig 6A**). This suggests that SITO-induced IL-1β production requires K^+^ efflux and caspase-1 activity, which have been shown to be essential for inflammasome activation and IL-1β release [32–34].

To further confirm the ability of SITO particles to induce inflammasome activation, treated cell lysates were measured for caspase-1 activation. After 12 and 24 h exposures to SITO, caspase-1 activation was significantly enhanced in cells when normalized to PBS controls (151.9% ± 10.2% at 12 h and 143.2% ± 8.9% at 24 h, mean ± SD) (**Fig 6B**). In **Fig 5**, IL-1β levels had only been measured as early as 24 h, so an ELISA was run on the 12 h media to determine whether caspase-1 activation by SITO led to IL-1β release by the 12 h time-point. However, as shown in **Fig 6C**, IL-1β was only significant for Min-U-sil treatment by 12 h, which caused caspase-1 activation at earlier times than SITO. Therefore, caspase-1 activation preceded IL-1β release.

### Inflammasome activation by ITO production particles in LPS-primed cells

**Fig 7:**
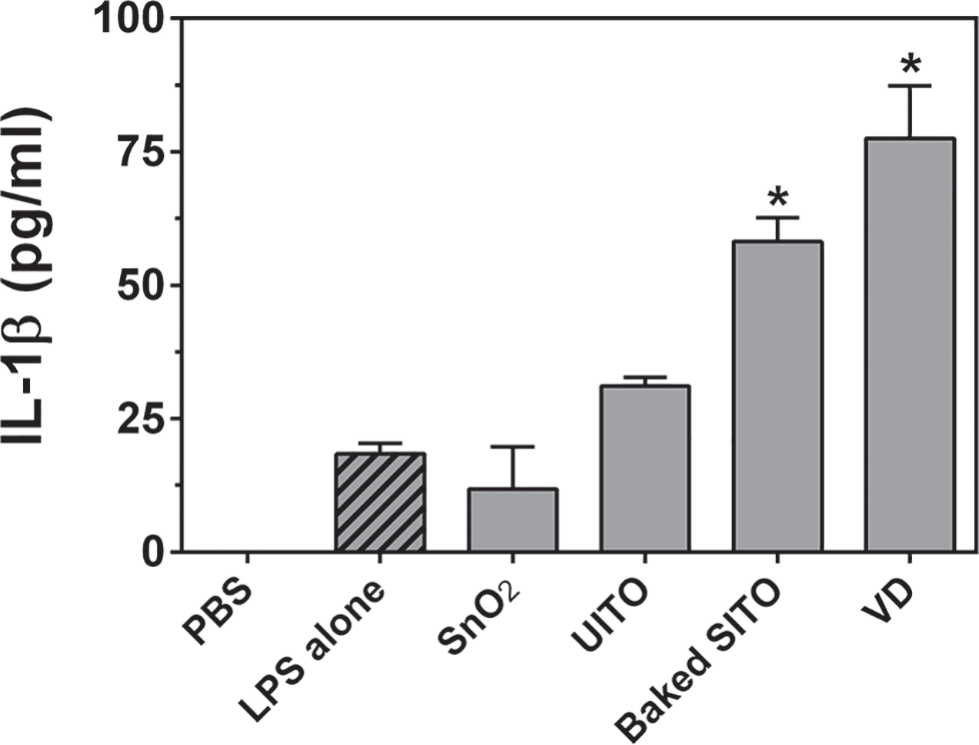
Particle-induced inflammasome activation in LPS-primed cells. RAW cells were primed with LPS for 3 h, followed by particle treatments for 24 h. Media were collected, and ELISAs were run to measure levels of IL-1β. Error bars represent the mean ± SD (n = 4). *, p < 0.05 compared to LPS priming alone.

As previously stated, the SITO particles collected in the workplace were contaminated with endotoxin. The presence of endotoxin accounts for the robust inflammasome activation observed, but we wanted to determine if endotoxin-free SITO particles could induce IL-1β release using the well-established method of priming macrophages with LPS prior to particle treatment. Baking SITO particles for 1 h at 270° C removed endotoxin activity (**Table 1**), and these “baked SITO” particles were used for further investigation. Given that these particles were generated post-sintering, which utilizes very high temperatures (>1000° C), baking them at 270° C is not expected to alter the physical or chemical characteristics. The other 3 particle samples, although not present with LPS in the workplace environment, could potentially activate the inflammasome in LPS-primed cells. Thus, all 4 particle samples (SnO_2_, UITO, baked SITO, and VD) were tested for their ability to induce IL-1β production in primed RAW cells. The results in **Fig 7** demonstrate that baked SITO and VD caused significant IL-1β production over LPS priming alone. Therefore, even without endotoxin contamination from the workplace, SITO was confirmed to be an inflammasome activator. VD was also shown to be an activator under these conditions. Given that VD particles were generated during indium recycling of leftover ITO materials, the VD particles may contain SITO.

### Baked SITO inflammatory effects on cells

**Fig 8:**
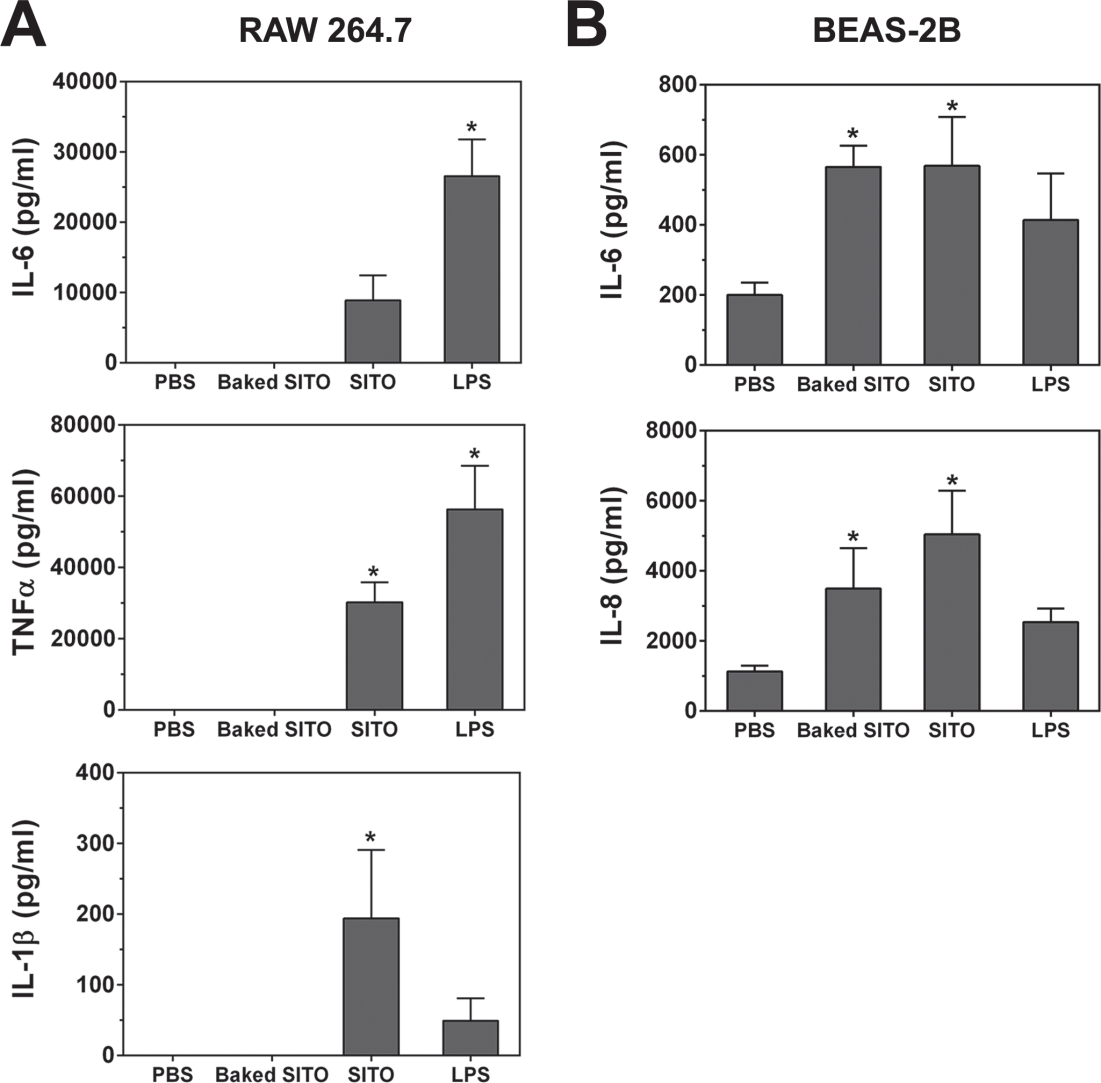
Cytokine production from baked SITO exposures. Cells were treated with SITO, baked SITO, or LPS for 24 h as in **Fig 5**. Media were collected, and ELISAs were run to measure levels of IL-6, TNFα, and IL-1β from RAW cells (**A**) or IL-6 and IL-8 from BEAS-2B cells (**B**). Error bars represent the mean ± SD (n = 4). *, p < 0.05 compared to PBS.

To further evaluate the adverse cellular effects of SITO exposure in the absence of endotoxin, cytokine release was re-measured with baked SITO, as shown in **Fig 8**. Baked SITO did not induce cytokine production from exposed (unprimed) RAW cells, indicating that the presence of endotoxin in the original SITO was necessary for the pro-inflammatory cytokine release in **Fig 5A**. However, the BEAS-2B cell results indicate that SITO particles caused significant production of IL-6 and IL-8 in the absence of endotoxin (**Fig 8B**). Therefore, the ability of baked SITO to induce pro-inflammatory responses *in vitro* appears to be cell type-specific.

**Fig 9:**
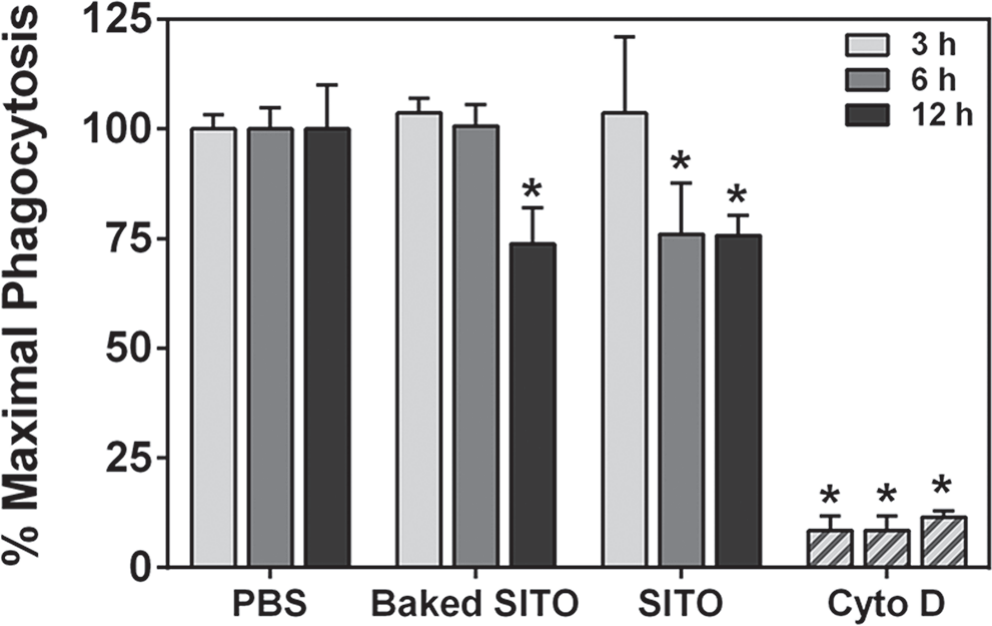
Baked SITO pre-treatment impairs phagocytosis. The phagocytosis assay was carried out as in **Fig 2**, but with baked SITO for comparison. Error bars represent the mean ± SD (n = 3). *, p < 0.05 compared to PBS at that time point.

Baked SITO still impaired phagocytosis by RAW cells, but the baking delayed the onset of this deficiency. As shown in **Fig 9**, the original, unbaked SITO exposure for 6 h led to significantly impaired phagocytosis of *E*. *coli* particles. However, when carried out with baked SITO, significant impairment was observed at 12 h. Thus, pathogen phagocytosis was still negatively impacted with endotoxin-free SITO exposure.

### Endotoxin-free SITO cytotoxicity

**Fig 10:**
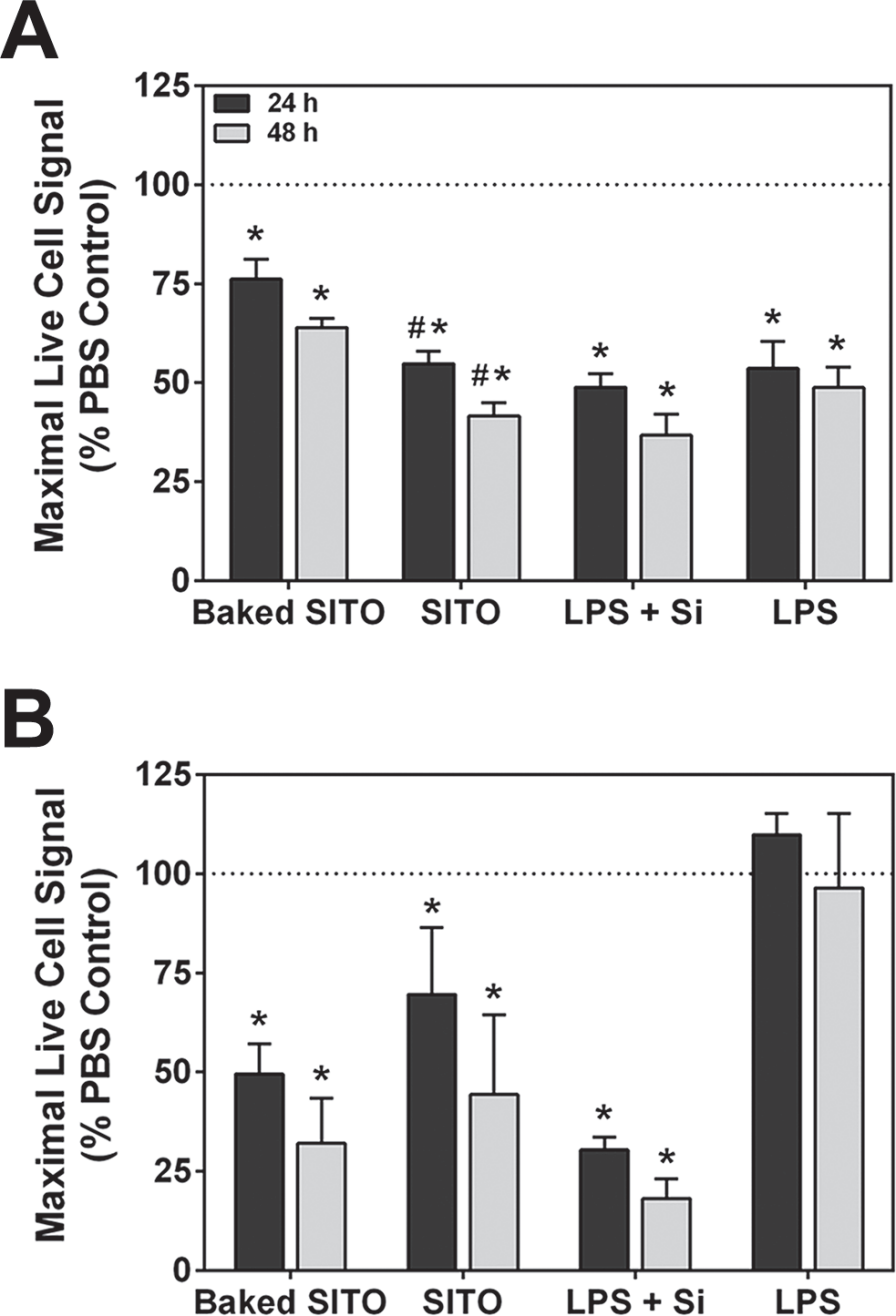
Baked SITO cytotoxicity in RAW and BEAS-2B cells. Treatments with SITO, baked SITO, LPS alone, or Min-U-sil (Si) were carried out for 24 and 48 h in RAW (**A**) and BEAS-2B (**B**) cells. MultiTox-Fluor Reagent (Promega) was added to all wells for 1 h at 37° C, and plates were read at 400ex/505em to determine the fluorescence signal from live cells. Values were normalized to PBS controls. Error bars represent the mean ± SD (n = 4). *, p < 0.05 compared to PBS.

A previous study by our laboratory showed the same SITO particles to be cytotoxic in both cell lines at 50 μg/ml [9]. However, it is possible that the presence of endotoxin contributed significantly to the cell viability loss measured. Therefore, a cytotoxicity assay was performed to measure the activity of a protease that is only active in viable cells. Compared to PBS controls, there were significant reductions in the live cell signal for both cell lines with baked SITO treatments (**Fig 10**). In RAW cells, the presence of endotoxin caused unbaked SITO to be significantly more toxic than baked SITO, but LPS was shown to be toxic as well in these cells (**Fig 10A**). This effect was not observed in BEAS-2B cells, as baked and unbaked SITO were equally toxic (**Fig 10B**). Regardless, the inactivation of endotoxin did not prevent cytotoxicity of SITO in either cell line, and therefore, SITO particles themselves were toxic to multiple cell types.

## DISCUSSION

The use of metal oxides in manufacturing has long been a concern for the occupational safety of exposed workers. With the boom of the consumer electronics industry in the United States and abroad, there has been a steady increase in the use of various metal-containing compounds. Indium-tin oxide represents one of the emerging materials being incorporated into many electronics and has been hazardous to workers in the ITO industry [5, 8, 35]. The present study aimed to evaluate the pro-inflammatory effects of particles collected from an ITO facility on cultured cells to uncover the molecular mechanisms of indium lung disease and determine if certain stages of ITO processing pose a higher risk for workers.

Recent studies have demonstrated inflammatory responses in the lungs of ITO-treated rodents [10, 12–14]. Thus, our findings that various indium-containing particles were capable of inducing cytokine production and macrophage dysfunction *in vitro* were consistent with prior studies. In humans, it is hypothesized that indium lung toxicity can lead to macrophage dysfunction, resulting in the excessive accumulation of alveolar surfactant proteins [8]. This has been seen in workers from the ITO industry that present with pulmonary alveolar proteinosis [8, 36] and in rodents exposed to ITO via inhalation [12, 13]. Therefore, macrophage dysfunction due to ITO particle uptake may be an initiating factor in pulmonary alveolar proteinosis in those workers. The results appear to be specific to particles that contain SITO (including VD from the reclaim department, where production waste and used SITO tiles are broken down) and not simply due to particle overload, as pre-uptake of SnO_2_ or UITO particles at the same concentration did not impair phagocytosis by RAW cells. Also, studies of other metal oxide-containing particles, such as steel-based welding fume particulates, did not impair RAW phagocytosis of *E*. *coli* [29], which further validates our interpretation of the results. It is interesting that VD appeared to be taken up the least by cells, and yet it impaired *E*. *coli* phagocytosis the most rapidly (**Figs 1 and 2**). Whether impaired phagocytosis has clinical implications in terms of increased susceptibility of workers to pulmonary infections is not known, but may warrant further investigation.

The examination of endpoints such as NFκB activation and cytokine release demonstrated that SITO was pro-inflammatory in both RAW and BEAS-2B cells. The robust release of IL-6, TNFα, and IL-1β by RAW cells treated with SITO suggested that toll-like receptor (TLR) and NFκB signaling may be occurring to induce “classical” macrophage activation. Release of IL-6 and IL-8 from BEAS-2B cells confirmed that this was not a cell type-specific response. Further, SITO was capable of inducing inflammasome activation and subsequent IL-1β release in unprimed macrophages. In similar studies, priming with LPS always preceded particle or ATP treatments to induce NLRP3 activation and IL-1β release [22, 23, 25, 37–40]. The cause of these pro-inflammatory findings was clearly due to endotoxin activity in the SITO particles. At first this finding was perplexing, as the process of sintering would inactivate any endotoxin present, due to the very high temperatures used. After consulting with an industrial hygienist who visited this specific ITO facility, we learned that post-sintered ITO tiles are sprayed with metalworking fluid during cutting and grinding as a coolant. As suspected, endotoxin activity was found in all of the metalworking fluids collected from the ITO department where these SITO particles were generated.

A strong advantage of this study is the utilization of particle samples that workers handle; thereby representing real-life exposures. Thus, the endotoxin-contaminated particles are still very much occupationally relevant, as these airborne dusts are what workers could be exposed to, as opposed to contaminant-free ITO purchased from a chemical supplier. If the SITO product was exposed to fluids containing endotoxin, those workers handling it (i.e., grinders) would be exposed not only to the airborne dusts, but also to endotoxin. In light of our findings regarding the synergistic effects of endotoxin, it is notable that half of the 10 clinical cases of indium lung disease reported in the medical literature as of 2010 occurred in ITO grinders, who would have the highest risk of a combined SITO-endotoxin exposure [8].

In addition, the fact that SITO required the presence of a TLR agonist (endotoxin contamination or LPS-priming) for significant IL-1β production agrees with all other studies of particle-induced NLRP3 inflammasome in macrophage cells, as referenced above. Activators of the inflammasome include a diverse variety of endogenous and exogenous particulates, and other occupational particles have been shown to induce the inflammasome as well [41]. This activation and subsequent pro-inflammatory cytokine release is thought to underlie progression of the particle-related diseases. For example, there have been several studies suggesting that silica- and asbestos-induced inflammasome activation in macrophages contribute to silicosis and asbestosis, respectively [22, 23, 25]. Similar to asbestos, various carbon nanotubes have also recently been shown to be NLRP3 agonists in LPS-primed human primary macrophages [42, 43]. Given that SITO and Min-U-sil induced similar levels of IL-1β release (**Fig 6**) and cell death (**Fig 10**) when the concentration of SITO was one-third that of Min-U-sil (50 vs. 150 μg/ml), SITO may be more potent than silica at initiating inflammatory lung injury *in vivo*. Although no other studies of indium-containing particle inflammasome activation exist in the literature, other metal particles have demonstrated the ability to activate the inflammasome. Caicedo et al. [44] found IL-1β production by human THP-1 macrophage cells exposed to Chromium, Cobalt, and Nickel metal ions.

Of course, it is of value to the field of toxicology and molecular biology to analyze the cytotoxicity and adverse responses of cells to this particle type without the influence of LPS. Therefore, assays were repeated with baked, endotoxin-free SITO. The contribution of the particle itself to cytokine release in BEAS-2B cells was significant, as baked SITO still induced IL-6 and IL-8 when ELISA assays were repeated (**Fig 8B**). The endotoxin-free particles were no longer pro-inflammatory in RAW cells; however, they still induced significant IL-1β release when cells were first primed with LPS. Baked SITO also impaired proper *E*. *coli* phagocytosis, but with a delayed response compared to unbaked SITO. Thus, in macrophage cells, endotoxin-free SITO particle exposure caused phagocytic dysfunction but did not induce cytokine release, while the exposure still led to a pro-inflammatory cytokine response in pulmonary epithelial cells.

Previous studies performed by our group to evaluate the toxicity of 8 different particles collected from the same ITO facility showed SITO to be the most cytotoxic to RAW and BEAS-2B cells [9]. After baking the same SITO sample, it was still found to cause significantly reduced viability in both cell lines (**Fig 10**). Gwinn et al. [11] observed similar findings in RAW cells treated with ITO particles, with elevated LDH activity beginning at 50 μg/ml, and Lison et al. [10] observed reduced cell viability in rat lung macrophage cells exposed to SITO using an LDH assay as well. Therefore, based on the work done by our lab and others, we conclude that ITO particles are cytotoxic to macrophages, which play a crucial role in taking up foreign particles and alerting the innate immune system to the presence of foreign particles.

It is important to note the difference in responses observed between unsintered and sintered ITO. After baking to remove endotoxin activity, the only difference among these samples was that SITO was subjected to high temperature and pressure (sintering), followed by grinding. SITO particles are slightly larger in terms of aerodynamic diameter (1.2 ± 0.8 μm SITO vs. 0.7 ± 0.6 μm UITO) and have reduced specific surface compared to UITO (3.0 ± 0.1 m^2^/g SITO vs. 4.4 ± 0.2 m^2^/g UITO). Therefore, the process of sintering creates some yet to be understood particle change(s) that causes cytotoxicity. This is evident based on the fact that baked SITO impaired phagocytosis, induced IL-1β release, and reduced viability in RAW macrophages, whereas UITO did not cause these changes.

The present study aimed to evaluate the adverse effects of various ITO production particles on cultured cells in an attempt to uncover potential mechanisms underlying indium lung disease. Although the four particle samples were readily taken up by both RAW and BEAS-2B cells, only those collected from post-sintering processes impaired phagocytic function and induced cytokine production. Overall, it appears that SITO may be the most hazardous to workers in this ITO production facility. This is partially due to the presence of endotoxin, which played a role in causing a robust inflammatory response in macrophages, and partially due to the sintered particle itself, which was cytotoxic to both cell lines and pro-inflammatory in BEAS-2B cells. Although SITO is the only ITO production particle in the current study expected to be contaminated with endotoxin in a real-life scenario, VD also demonstrated the potential to induce the inflammasome. Thus, risk in this facility is not limited to a single process or material.
